# Intervention Strategies using *Agada* (Ayurvedic Antitoxic Formulations) for Diseases caused by Endocrine Disruptors

**DOI:** 10.2174/0118715303426053260413072002

**Published:** 2026-04-30

**Authors:** Nilima Wadnerwar

**Affiliations:** 1 Department of Agadtantra, Mahatma Gandhi Ayurved College, Hospital & Research Centre, Salod (H), Datta Meghe Institute of Higher Education and Research (Deemed to be University), Wardha, Maharashtra, India

**Keywords:** *Dushivisha*, endocrine system, free radical, *Garavisha*, oxidative stress, clinical trials

## Abstract

**Introduction:**

Chemicals consumed through food, water, air, and personal care products disrupt our endocrine system, contributing to a range of life-threatening diseases. Since this toxicity is cumulative, it is essential to incorporate the principles of toxicity treatment. The review was conducted to evaluate the potential utility of Agada formulations in managing diseases associated with endocrine-disrupting chemicals.

**Methods:**

Electronic searches were performed using terms “Agada”, “Ayurveda”, “antitoxic”, “visha”, “dūṣhivīṣha”, “endocrine-disrupting chemicals”, and “toxicity” including animal, clinical, or pharmacological studies of Agada formulations in any toxicant or cumulative toxin model; or conceptual reviews linking Agada to environmental/chemical toxicity. By reviewing Ayurvedic literature, studying research on *Agada*, and correlating the principles of treatment of *dushivisha*, this article examines the potential of Ayurvedic treatment for diseases induced by endocrine disruptors.

**Results:**

The conceptual framework of *Dūṣhivīṣha* provides a strong philosophical parallel to chronic low-dose EDC exposure, legitimizing research interest. Several formulations exhibit general antitoxic effects in chemical or drug toxicity models primarily mediated by antioxidant and anti-inflammatory mechanisms.

**Discussion:**

Agada formulations may offer an integrative approach to mitigating EDC-related diseases. Their known pharmacological actions, such as antioxidant activity, free radical scavenging, Nrf2 upregulation, NF-κB inhibition, and adaptogenic effects, align with the major pathophysiological pathways triggered by EDCs. However, the current evidence base is limited by the absence of standardized formulations, inconsistent dosing, lack of endocrine biomarker assessment, and a scarcity of clinical trials.

**Conclusion:**

By applying the treatment principles of Garavisha (swallowed poison) and Dushivisha (latent poison), disorders resulting from endocrine disruptors can be effectively addressed using Agada directing to generate empirical evidences through clinical trials.

## 1 INTRODUCTION

The endocrine system is a complex network of internal organs that produce hormones that regulate and maintain homeostasis in the human body. Endocrine-disrupting chemicals (EDCs) are exogenous substances that interfere with the normal functioning of the endocrine system [1]. Humans are exposed to thousands of such chemicals throughout their lifetime *via* food, water, air, and soil. These substances present in the environment (air, soil, and water), food sources, personal care products, and various manufactured goods can disrupt hormonal balance and lead to significant health issues.

EDCs are commonly found in everyday items such as plastic bottles, metal food cans, detergents, flame retardants, toys, cosmetics, pesticides, and processed foods [2]. They can enter the body through ingestion, inhalation, or dermal absorption. Once inside the body, these chemicals can cause a wide range of adverse effects, including developmental, reproductive, neurological, and immunological disorders.

Currently, more than 350,000 chemicals and chemical mixtures are registered for commercial production and use globally, up to three times higher than commonly estimated [3]. Each year, hundreds of new chemicals enter the market, yet only a small fraction have been adequately tested for safety. Of particular concern are chemicals that persist in the environment, contaminate air, water bodies, and soil, enter the food chain and drinking water, or bio-accumulate in human and animal tissues.

According to the World Health Organization, exposure to selected chemicals was responsible for an estimated 1.6 million deaths in 2016. Furthermore, a report by the National Toxicology Program indicated that even very low doses of hormone-like substances can disrupt physiological functions in test animals. These findings highlight an urgent need to develop effective prevention and intervention strategies to control and manage diseases linked to Endocrine-Disrupting Chemicals (EDCs).

Ayurvedic toxicology (*Agadatantra*) emphasizes the use of antitoxic formulations known as *Agada*, traditionally employed in the treatment of poisoning and its complications, as well as for various other therapeutic indications. Ancient texts describe “*dūṣivīṣa*,” latent toxins accumulating over time, paralleling chronic environmental chemical exposure [4]. Agada formulations, traditionally polyherbal or mineral preparations, are reputed to neutralize or expel poisons while restoring *dosha* balance. This review explores and advocates integrating Ayurvedic antitoxic strategies, specifically Agada formulations, as a potential intervention for the management of disorders caused by endocrine disruptors. The present study aims to review and explore the existing evidence on the efficacy, mechanisms, and safety of Ayurvedic Agada formulations for conditions caused by or related to EDC exposure.

## 2 MATERIALS AND METHODS

Data for this study were collected from classical Ayurvedic treatises, standard textbooks on *Agadatantra* (Ayurvedic toxicology), and peer-reviewed journal articles. An extensive manual literature review was conducted using various Ayurvedic and *Agadatantra* references.

Electronic searches were performed (PubMed, AYUSH Research Portal, Google Scholar, AYU Journal, *JAIMS*, *IRJAY*, and *Ayurline*) through October 2025 using terms: “Agada”, “Ayurveda”, “antitoxic”, “visha”, “dūṣivīṣa”, “endocrine-disrupting chemicals”, and “toxicity”. Quality appraisal followed PRISMA-scoping standards.

### 2.1 Inclusion Criteria

Studies describing Agada formulations with experimental/clinical evaluation in toxic models and reviews conceptually linking Agada or visha management to modern chemical toxicity were included in the study. As there are very few research papers on Agada, articles published from 2016 to 2025 are included.

### 2.2 Exclusion Criteria

Non-Ayurvedic detox regimens, editorials without pharmacological or conceptual content, and studies solely on individual herbs without reference to Agada formulation were excluded from the study.

### 2.3 Data Extraction

For each study: formulation name, experimental model, toxicant type, outcome measures, and inferred endocrine relevance. Data were synthesized narratively due to heterogeneity.

### 2.4 Quality Assessment

Modified CAMARADES checklist for preclinical studies; methodological transparency for conceptual reviews. PRISMA flow is summarized below.

The collected data were systematically segregated, edited, consolidated, and critically reviewed to evaluate the role and scientific relevance of various *Agada* formulations in managing diseases associated with endocrine disruptors. By examining classical Ayurvedic texts, analyzing contemporary research on *Agada*, and correlating these findings with the principles of *Dushivisha* (cumulative toxicity) treatment, this review explores the potential of Ayurvedic approaches in addressing diseases caused by endocrine-disrupting chemicals.

## 3 RESULTS

132 articles were identified through a database search. After removal of duplicates (27), 105 titles and abstracts were screened. On withdrawal of 81 non-AGADA and irrelevant articles, 24 full-text articles were assessed and included in this study.

### 3.1 Exposure to Endocrine-Disrupting Chemicals (EDCs)

Humans are exposed to endocrine-disrupting chemicals through several major pathways, including inhalation of contaminated air and particles, ingestion of polluted food and drinking water, and direct dermal contact [5]. These environmental exposures are of growing concern due to their potential long-term health impacts, many of which remain unknown. Consumer products also represent a significant source of EDC exposure, including items such as plastics, cosmetics, and household chemicals.

In response to evidence of widespread exposure and associated health risks, certain EDCs have been restricted in some countries. These chemicals can interfere with any hormone-regulated system in the body, with effects dependent on the timing of exposure, dosage, and the specific type of endocrine disruptor involved.[6]

### 3.2 Health Manifestations Associated with EDC Exposure

Various dietary and environmental sources, such as red meat, processed and refined foods, caffeine, dairy products, pesticides, and cosmetics, may contain endocrine-disrupting chemicals. EDC exposure has been linked to a wide range of health conditions, including breast and prostate cancer, endometriosis, infertility, diabetes, metabolic syndrome, early puberty, obesity, increased susceptibility to infections, autoimmune diseases, asthma, cardiovascular disease, hypertension, stroke, Alzheimer’s disease, Parkinson’s disease, and neuro developmental disorders such as ADHD and learning disabilities (Table **1**) [7].

Clinical signs of endocrine disruption may include abdominal obesity, purple striae (stretch marks) on the skin, a rounded face and neck, elevated blood pressure, fatigue, muscle weakness, mood swings, and depression.

To minimize exposure to EDCs, it is advisable to avoid using plastic containers, bottles, and packaging. Canned foods and beverages should also be limited. A diet emphasizing fresh, organic, and minimally processed foods is recommended as a preventive strategy [8].

### 3.3 Mechanism of Action of Endocrine Disruptors

Endocrine-Disrupting Chemicals (EDCs) can induce adverse developmental, reproductive, neurological, and immunological effects in both humans and wildlife. These substances act through various mechanisms, including mimicking naturally occurring hormones such as estrogens and androgens, antagonizing hormone receptors, blocking normal hormonal binding, altering the synthesis and degradation of endogenous hormones, and modulating hormone receptor levels.

EDCs induce oxidative stress, inflammation, and receptor dysregulation [23]. Many *Agada* ingredients—*Haridra* (*Curcuma longa* Linn.) [24], *Guduchi* (*Tinospora cordifolia* Wild) [25], *Amalaki* (*Emblica officinalis* Linn.) [26] possess ROS scavenging and detox enzyme up-regulation (Nrf2 pathway), anti-inflammatory and NF-κB inhibition, as well as Potential endocrine modulation (phytoestrogenic or adaptogenic).

These mechanisms plausibly intersect with EDC pathogenesis (thyroid, reproductive, and metabolic dysfunction).

### 3.4 Endocrine Disruptors in the Context of Ayurveda

Ayurvedic toxicology provides insightful parallels to modern understanding of EDCs through the concepts of *Dushivisha* [27] and *Garavisha* [28]. *Dushivisha* refers to a form of cumulative or latent toxicity caused by repeated exposure to low-potency toxins or small doses of certain substances over time. Though initially non-lethal, these toxins accumulate in the body, provoking delayed or chronic pathological responses.

*Garavisha* is described as an artificial poison, formed by the combination of two or more non-toxic substances often ingested unknowingly through food, drinks, or topical applications that become toxic when combined.

These concepts provide a traditional foundation for understanding the cumulative and insidious effects of modern-day endocrine disruptors.

### 3.5 Poisoning and the Role of Free Radicals

When toxins enter the body, they can initiate the formation of free radical unstable atoms or molecules with one or more unpaired electrons. These reactive species damage cellular components, contributing to various disease processes [29]. Free radicals can arise from both endogenous and exogenous sources.

Endogenous causes include enzyme deficiencies and oxidative stress within the body. Exogenous sources include exposure to harmful chemicals, pesticides, smoking, alcohol, and consumption of certain foods such as refined sugar, processed carbohydrates, soft drinks, white bread, potatoes, red and processed meats, and unhealthy cooking oils [30].

Additionally, psychological factors such as desire (*Kama*), anger (*Krodha*), greed (*Lobha*), delusion (*Moha*), jealousy (*Irshya*), anxiety (*Chinta*), fear (*Bhaya*), and grief (*Shoka*) are recognized in Ayurveda as contributors to oxidative stress and free radical formation. Chronic stress, driven by lifestyle irregularities and poor dietary habits, further exacerbates the production of free radicals, ultimately leading to lifestyle-related disorders.

### 3.6 Correlating *Dushivisha* with Endocrine Disruptors

There are significant conceptual similarities between *Dushivisha* in Ayurveda and the mechanism of harm posed by endocrine-disrupting chemicals:

#### 3.6.1 Chronic Accumulation

Both involve prolonged, low-level exposure to harmful substances that accumulate in the body over time, leading to long-term health consequences.

#### 3.6.2 Systemic Effects

*Dushivisha* is associated with various chronic conditions, similar to how EDCs are implicated in a wide range of disorders across multiple physiological systems.

#### 3.6.3 Exposure Pathways

The sources and pathways of exposure are closely aligned. Both originate from contaminated food, water, and environmental pollutants.

#### 3.6.4 Hormonal Disruption

*Dushivisha* is said to disturb the body’s natural balance (*dosha*) and physiological functions, analogous to the hormonal disruption caused by EDCs.

#### 3.6.5 Persistent Nature

Both are difficult to eliminate from the body due to their bioaccumulative and persistent nature.

Diseases caused by endocrine disruptors exhibit a pathogenesis that closely resembles that of *Dushivisha* or *Garavisha*. Hence, Ayurvedic management protocols traditionally used for these toxic conditions, especially involving *Agada* (antidotal therapy), can be effectively applied.

#### 3.6.6 Intervention Strategy through Ayurvedic Treatment

Management of *Dushivisha* includes Fomentation (*Swedana*) followed by *Shodhana Karma* (bio-purification procedures), tailored according to the patient’s *Prakriti* (constitution), age, strength, stage of disease, and seasonal factors. After proper purification of the body, daily administration of *Dushivishari Agada* is indicated [31].

**Fig. (1):**
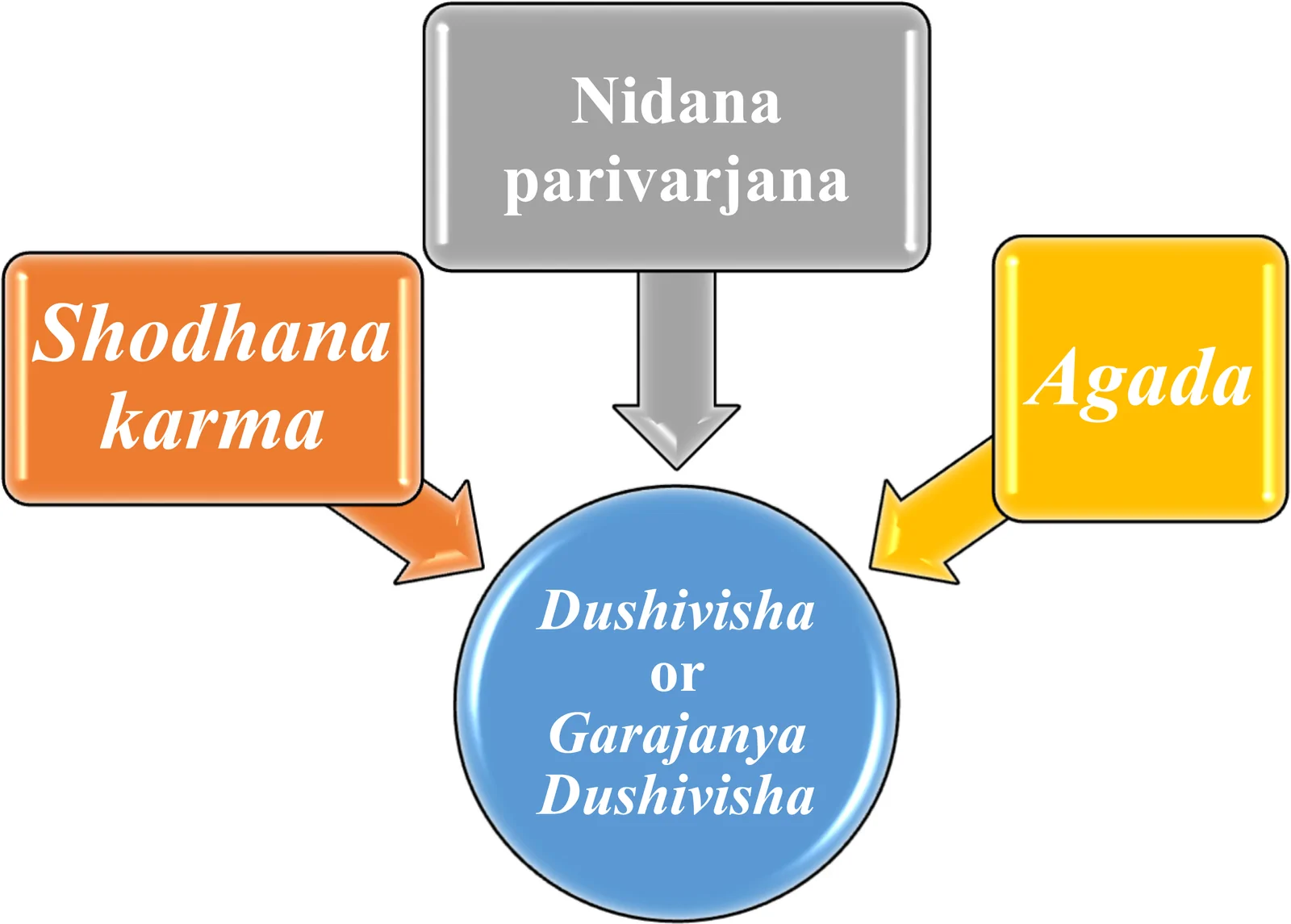
Treatment strategy for *Dushivisha* or *Garavisha.*

Procedures such as *Vamana* (therapeutic emesis), *Virechana* (purgation), *Raktamokshana* [32] (bloodletting), and *Nasya* (nasal medication) are employed to eliminate accumulated toxins (Fig. **1**).

The most crucial principle is *Nidana Parivarjana,* avoiding causative factors. Preventing further exposure to toxic substances is fundamental to both treatment and long-term prevention.

For managing diseases caused by endocrine disruptors, the comprehensive Ayurvedic treatment protocol includes:

***Nidana Parivarjana*** – Elimination of the root cause or exposure.

***Shodhana Karma*** – Detoxification to remove accumulated toxins.

***Agada Prayoga*** – Use of *Agada* (antitoxic formulations) with specific properties.

**Fig. (2):**
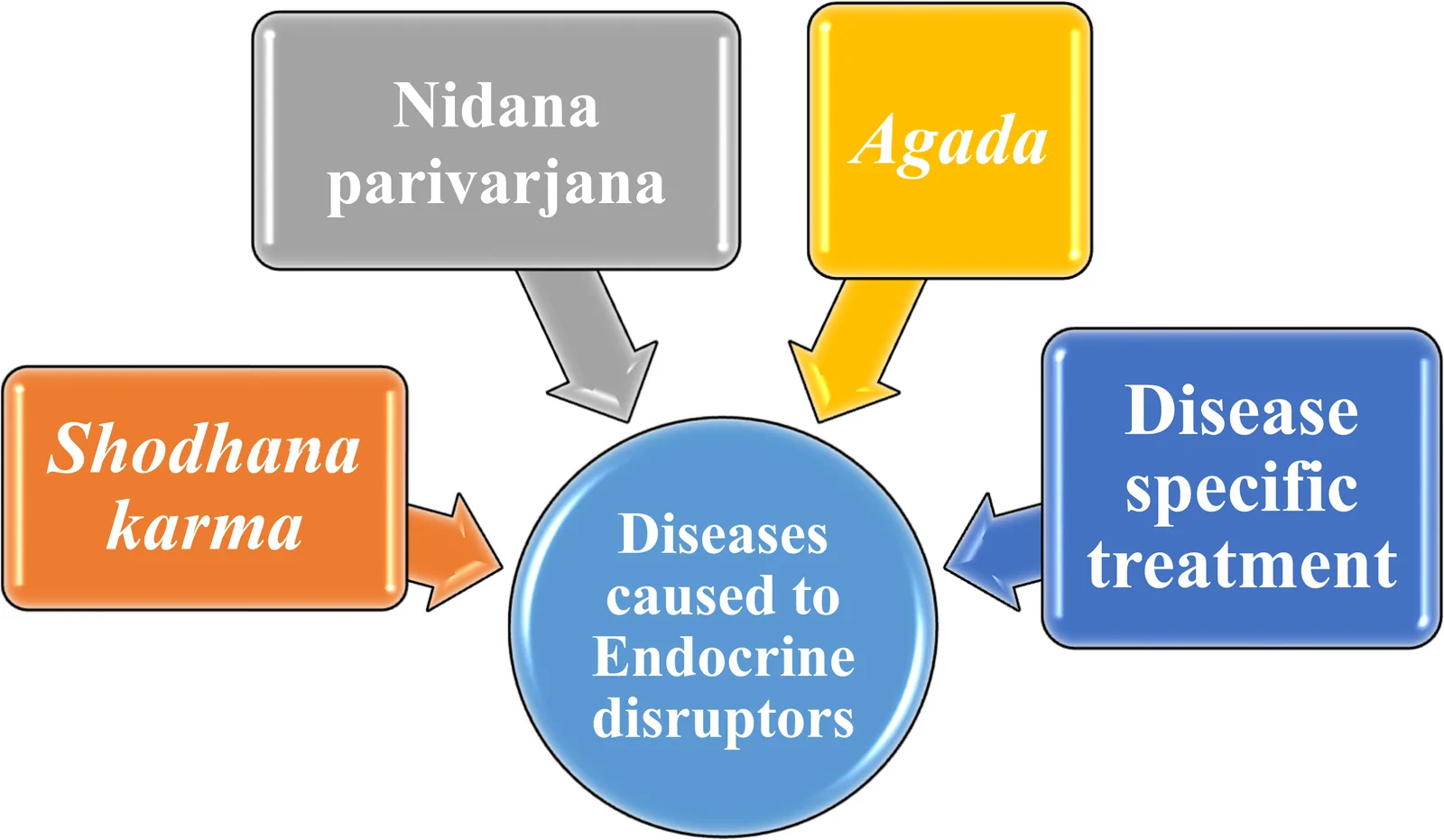
Treatment strategy for diseases caused by endocrine disruptors.

***Vyadhi-specific Chikitsa*** – Disease-specific therapeutic interventions as described in Ayurveda (Fig. **2**).

### 3.7 Selection of *Agada* (Anti-toxic Formulation)

The *Agada* formulations employed in the management of poisoning must possess specific therapeutic properties, including the ability to neutralize the toxic effects of poisons, purify the blood, balance all three *Dosha* (body constituents), remove pathological obstructions from vital organs, and safeguard the heart from toxic damage. The constituents of *Agada* should exhibit antioxidant activity and free radical scavenging properties to effectively mitigate oxidative stress induced by toxic substances.

*Agada* has significant potential for the treatment of poisoning and various other disease conditions and infections. In general, anti-toxic formulations incorporate herbs categorized under *Vishaghna Mahakashaya*, such as *Shirisha* (*Albizia lebbeck* Linn.), *Haridra* (*Curcuma longa* Linn.), *Tagara* (*Valeriana wallichii* DC.), *Lodhra* (*Symplocos racemosa* Roxb.), *Vacha* (*Acorus calamus* Linn.), and *Daruharidra* (*Berberis aristata* DC.). Many of these herbs are recognized for their blood-purifying properties. Several of the ingredients also demonstrate antioxidant, anti-inflammatory, analgesic, antihistaminic, and anti-allergic effects (Table **2**).

## 4 DISCUSSION

### 4.1 Endocrine Disrupting Chemicals (EDCs) and the Role of *Agada* Formulations

Endocrine disruptors are exogenous substances that are not naturally present in the human body. These compounds interfere with the endocrine system by disrupting the production, release, transport, metabolism, binding, action, or elimination of the body's endogenous hormones. Over the past few decades, exposure to EDCs has markedly increased, contributing to a rising prevalence of hormone-related disorders and various lifestyle-associated diseases.

Combating EDC health effects requires prevention of exposure (Nidan parivarjana), elimination of harmful chemicals or toxins (Ama), normalization of body constituents (Dosha), and reversal of health (*Swasthya*).

*Shodhana Karma* (detoxification) in Ayurveda offers profound therapeutic benefits by eliminating accumulated toxins (*ama*) and imbalanced *doshas* from the body, leading to comprehensive physical and mental rejuvenation. Its primary effects include disease treatment and prevention, enhanced metabolism, and overall systemic balance. [47]. These procedures also have a positive effect on antioxidant levels. [48]

In Ayurvedic medicine, *Agada* [38] formulations originally indicated for different types of poisoning may offer therapeutic potential in conditions associated with EDC exposure. Selection of *Agada* is described in Table **2** exclusively on the basis of their indications and the pharmacological research conducted on the *Agada* or the individual contents of *Agada*.

Classical *Agada*s such as Brahma Agada, Trimurti Agada, Maheshwara Agada, Manjishthadi Agada, Dhooma Agada, Gandhahasti Agada, Ksharagada, Himavana Agada, Lodhradi Agada, Prajapatya Agada, Shleshmatakadya Agada, and Sarva Vishanashaka Agada are indicated for all types of toxic exposures. They can be administered in animate, inanimate, and artificial poisoning and chronic toxicity. Hence, they can be repurposed for diseases linked to EDCs.

Similarly, *Agada* indicated in *Dushi Visha* (accumulated or latent toxins) and *Gara Visha* (artificially created poisons), such as *Churna Agada*, *Dushivishari Agada*, *Pippalyadi Agada*, and *Murvadi Agada*, are also relevant in managing EDC-induced pathologies.

### 4.2 Evidence-based Applications of *Agada* Formulations

*Sindhuvaradi Agada* has demonstrated efficacy in reducing CPK-MB and SGOT levels, maintaining lipid profiles, and minimizing cardiac myocyte damage in Wistar rats with doxorubicin-induced cardiotoxicity [38]. This suggests its potential use in managing EDC-induced cardiac dysfunction.

*Siddhartakadi Agada*, which has been shown to be efficacious for malarial fever [38], may also show promise in managing infectious diseases, particularly those exacerbated by EDC exposure.

*Bilwadi Agada* exhibits nephroprotective activity in gentamicin-induced nephrotoxicity in male Wistar rats. It is also observed to reduce Cypermethrin-induced nephrotoxicity (rat) by reducing creatinine levels and providing histologic protection. [44] Additionally, it alleviates symptoms such as burning, pain, and inflammation in scorpion bites and has demonstrated antimicrobial and antifungal activity at higher concentrations [38].

*Dashanga Agada* has shown anti-inflammatory effects in carrageenan-induced inflammation models, as well as antimicrobial and free radical-scavenging properties. It is also effective in relieving burning, pain, and inflammation associated with scorpion envenomation [38].

*Dushivishari Agada* has a cytoprotective effect on ovaries and follicles in MSG-induced reproductive toxicity. *Dushivishari Agada* is more efficacious than cetirizine for the management of Urticaria [38]. In an animal model, *Dushivishari Agada* was found to prolong the nadir times of Hb, WBC, and R.B.C., with earlier recovery, compared with Carboplatin [45]. It may contribute to reducing the chemotoxic effects of Carboplatin in patients with cancer. It also exhibits inhibitory activity against Butyryl cholinesterase using Ellman’s method for Alzheimer’s disease [46]. *Dushivishari Agada* has effectively reduced the weight and height of fetuses in mice exposed to teratogenic agents. Given these multifaceted properties, *Dushivishari Agada* holds potential as a therapeutic agent in conditions associated with EDC exposure, including Alzheimer’s disease, breast and prostate cancers, endometriosis, and infertility. Additionally, *Sitadi Agada* and *Siddhartakadi Agada*, both indicated in mental health disorders, may be beneficial in EDC-related cognitive impairments such as Alzheimer’s disease.

Hence, *Dushivishari Agada* may be prescribed for Alzheimer's disease, Breast/prostate cancer, Endometriosis, and Infertility caused by E.D.C. *Sitadi Agada* and *Siddharthakadi Agada* may be efficacious in Alzheimer's disease caused by E.D.C., as they are indicated in mental disorders.

### 4.3 Dosage and Administration

*Agada* formulations are generally administered orally, with honey or ghee as an adjuvant (*anupana*), both of which possess intrinsic antitoxic properties. However, standardized dosing guidelines for *Agada* formulations are not well-established in classical texts. Dosage must be tailored to individual factors, including the patient’s age, body weight, clinical condition, and the treating physician’s clinical judgment. To ensure optimal efficacy, especially in acute poisoning scenarios, systematic dose-escalation studies are needed. Although some preliminary investigations have been conducted within the field of Ayurveda, more rigorous clinical and pharmacological research is essential to validate and standardize the use of *Agada* formulations in contemporary toxicological conditions.

The evidence found relates to preclinical and pharmacological research. However, the absence of human trials and endocrine biomarkers, and variability in formulation composition and dose, may be the lacunae in the present scenario.

Hence, it is recommended to test standardized *Agada* in EDC models (*e.g.*, BPA, DEHP, PFAS) and assess hormonal, oxidative, and reproductive outcomes. It is required to conduct clinical trials measuring EDC biomarkers, thyroid and reproductive hormones, and oxidative indices, with Agada as the main drug or as an adjuvant, using a reverse pharmacological approach.

## CONCLUSION

*Agada* formulations described in Ayurvedic toxicology may be effectively utilized to manage diseases associated with Endocrine-Disrupting Chemicals (EDCs), particularly by adopting the therapeutic approach outlined for *Dushivisha* (latent toxicity). To substantiate this intervention strategy and ensure its clinical applicability, further well-designed clinical trials and dose-escalation studies are essential. Such research is necessary to validate efficacy, establish standardized dosing protocols, and generate robust scientific evidence supporting the integration of *Agada* into contemporary toxicological and endocrine disorder management.

*Agada* formulations are promising traditional antitoxic agents with pharmacological properties (antioxidant, immunomodulatory, adaptogenic) potentially relevant to EDC-induced diseases. However, it is essential to generate direct empirical evidence demonstrating efficacy in endocrine disorders caused by EDCs. Integrative, mechanistic, and translational studies are crucial to substantiate Ayurvedic detoxification within modern endocrine toxicology.

## Figures and Tables

**Table 1:** Common EDCs, their uses, and effects.

**Common EDCs**	**Used In**	**Effects**
DDT	Agriculture and malaria control, Pesticides	On estrogen receptors, it disrupts both male and female reproductive organs by influencing the development *via* disruption of multiple endocrine pathways [9, 10]
Cadmium	Tobacco smoke	Exposure during pregnancy causes decreased birth weight and premature births [11]
Bisphenol A (BPA), Phthalates, Phenol	Plastics and Food Storage Materials	Obesity, brain diseases, cancer, asthma, heart disease, disruption of the hormone system, and reproductive system [12]
Atrazine	Herbicide to prevent the growth of weeds	Affects both male and female reproductive organs. Prolonged estrogen cycle, swelling of luteal cells, decrease in ovary and uterine weight in females, decrease in body weight, testosterone, increase in estradiol in males [13].
Bitertanol	Fungicide, used for fruit diseases, banana, peanut leaf spot diseases, rust, and mildews on a variety of crops	Inhibit aromatase activity, estrogen production, and increase in androgens [14].
Polychlorinated biphenyls (PCBs) and Dioxins	Industrial Solvents or Lubricants and their Byproducts	Skin and liver problems, elevated lipid levels [15].
Polybrominated biphenyl ethers (PBDEs)	Manufactured products to reduce the chances of catching fire	Effect on thyroid, liver, and brain development [16].
Brominated Flame Retardants	Electronics and Building Materials	Endocrine disruption and thyroid dysfunction [17].
Phthalates, Parabens, UV Filters	Personal Care Products, Medical Tubing, Sunscreen	Disturb progesterone and hormone homeostasis, and further, pubertal development [18].
Triclosan	Anti-Bacterial Soaps, Colgate Total	Allergy and sensitivity to certain foods, bacterial resistance [19].
Chlorpyrifos	Pesticides	affects the nervous system [20].
2, 4-D, Glyphosate	Pesticides	Salt forms of 2,4-D can cause severe eye and skin irritation [21].
Lead, Phthalates	Children's Products	Reproductive and developmental toxicity, potential genotoxicity [22].

**Table 2:** Selection of *Agada.*

**S. No.**	**Indications**	**Anti-toxic Formulation** (***Agada)***
1	*Agada,* which are indicated in all types of poisoning	*Bramha Agada, Maheshwara Agada, Himavana Agada, Lodhradi Agada, Prajapatya Agada* [33], *Trimurti Agada* [34], *Manjishthadi Agada* [35], *Dhooma Agada, Gandhahasti Agada, Ksharagada, Sarva Vishanashaka Agada* [36], *Shleshmatakadya Agada* [37].
2	*Agada,* which are indicated in *Dushivisha* and *Garavisha*	*Churna Agada, Dushivishari Agada* [38], *Pippalyadi Agada* [39], *Murvadi Agada* [40]
3	Cardiac diseases	*Sindhuvaradi Agada* [38, 41], *Dashanga Agada* [42]
4	Infectious diseases	*Siddhartakadi Agada* [38], *Bilwadi Agada* [38, 43, 44], and *Dashanga Agada* [38, 42]
5	Breast/prostate cancer, Endometriosis, Infertility	*Dushivishari Agada* [38, 45]
6	Alzheimer disease	*Dushivishari Agada* [46], *Sitadi Agada* [38], *Siddharthakadi Agada* [38]

## LIST OF ABBREVIATIONS

EDC: Endocrine Disrupting Chemicals
ADHD: Attention Deficit Hyperactivity Disorder
DDT: Dichloro Diphenyl Trichloroethane
UV: Ultraviolet
CPK MB: Creatine Kinase MB
SGOT: Serum Glutamic Oxaloacetic Transaminase
Hb: Hemoglobin
WBC: White Blood Cell
RBC: Red Blood Cell
